# ATR-Mediated FANCI Phosphorylation Regulates Both Ubiquitination and Deubiquitination of FANCD2

**DOI:** 10.3389/fcell.2020.00002

**Published:** 2020-02-04

**Authors:** Winnie Tan, Sylvie van Twest, Vincent J. Murphy, Andrew J. Deans

**Affiliations:** ^1^Genome Stability Unit, St Vincent’s Institute of Medical Research, Fitzroy, VIC, Australia; ^2^Department of Medicine (St Vincent’s Hospital), The University of Melbourne, Melbourne, VIC, Australia

**Keywords:** FANCI, phosphorylation, FANCD2, ubiquitination, deubiquitination

## Abstract

DNA interstrand crosslinks (ICLs) are a physical barrier to replication and therefore toxic to cell viability. An important mechanism for the removal of ICLs is the Fanconi Anemia DNA repair pathway, which is initiated by mono-ubiquitination of FANCD2 and its partner protein FANCI. Here, we show that maintenance of FANCD2 and FANCI proteins in a monoubiquitinated form is regulated by the ATR-kinase. Using recombinant proteins in biochemical reconstitution experiments we show that ATR directly phosphorylates FANCI on serine 556, 559, and 565 to stabilize its association with DNA and FANCD2. This increased association with DNA stimulates the conjugation of ubiquitin to both FANCI and FANCD2, but also inhibits ubiquitin deconjugation. Using phosphomimetic and phosphodead mutants of FANCI we show that S559 and S565 are particularly important for protecting the complex from the activity of the deubiquitinating enzyme USP1:UAF1. Our results reveal a major mechanism by which ATR kinase maintains the activation of the FA pathway, by promoting the accumulation of FANCD2 in the ubiquitinated form active in DNA repair.

## Introduction

Many chemotherapeutic drugs kill cancer cells by inducing toxic DNA interstrand crosslinks (ICLs). ICLs prevent DNA strand separation and therefore stall DNA transcription and replication complexes (Deans and West, 2011). A critical step in the repair of replication forks stalled by ICLs is the biochemical modification of FANCD2 protein by mono-ubiquitination. Genetic deficiency in this pathway leads to Fanconi anemia (FA), characterized by hypersensitivity to DNA crosslinking agents, bone marrow failure, infertility, and cancer predisposition (Garaycoechea and Patel, 2014; Tsui and Crismani, 2019).

FANCD2 monoubiquitination is temporally and spatially controlled at stalled forks by the FA core complex (a RING E3 ligase), USP1:UAF1 (a deubiquitinating enzyme) and ATR kinase (Ishiai et al., 2017). FANCI is the heterodimeric partner of FANCD2 and plays an essential role in this regulation. In particular, FANCI itself is a target for monoubiquitination by the FA core complex (Smogorzewska et al., 2007), is a substrate of ATR kinase (Chen et al., 2015) and contains a USP1:UAF1 binding site necessary for deubiquitination of FANCD2 (Cohn et al., 2009). FANCD2 associates with FANCI as a heterodimer during ICL repair to signal DNA repair proteins that contain ubiquitin binding motifs, to promote DNA repair via homologous recombination or translesion synthesis (Smogorzewska et al., 2010; Klein Douwel et al., 2014).

A crystal structure of FANCI:FANCD2 complex revealed that the lysine residues targeted for monoubiquitination in FANCI (K522 in mouse corresponding to K523 in human) and FANCD2 (K559 in mouse corresponding to K561 in human) are embedded in the dimer interface (Joo et al., 2011). This raised questions regarding the accessibility of ubiquitin and E3 enzyme into the dimer during the conjugation reaction. In contrast, several validated ATR kinase sites of FANCI (S556, 559, and 565) within an ST/Q cluster (Chen et al., 2015; Cheung et al., 2017) were found to be exposed on the FANCI surface adjacent to the heterodimer interface (Joo et al., 2011). As ATR kinase activity is required for optimal FANCD2 monoubiquitination (Shigechi et al., 2012), it was proposed that ATR phosphorylation of FANCI occurs prior to FANCI:FANCD2 ubiquitination to promote a partial opening of the FANCI:FANCD2 complex, and access to the ubiquitin ligase complex. Support for this comes from the observation that FANCI phosphorylation at serine sites 559 and 565 occurs predominantly on the monoubiquitinated form (Cheung et al., 2017). The UAF1-binding SIM domain of FANCI is next to serines 559 and 565, so an alternative explanation is that ATR acts on these sites post-monoubiquitination, to prevent USP1:UAF1 binding. In this manner, phosphorylation would prevent deubiquitination by USP1. Indeed, phosphomimetic FANCI-S559D/S565D promotes partial cellular resistance to the USP1 inhibitor ML323 (Cheung et al., 2017), but the complication in living cells is that inhibition of deubiquitination actually prevents the correct localization of FANCD2 to new DNA breaks (Castella et al., 2015). The complex intersection of phosphorylation, monoubiquitination, and deubiquitination in regulation of the FA pathway requires investigation using a defined biochemical system.

We previously reconstituted the *in vitro* monoubiquitination and de-ubiquitination of FANCI:FANCD2 using purified proteins. Maximal monoubiquitination required the FANCB-FANCL-FAAP100 (BL100 enzyme module) and FANCC-FANCE-FANCF (CEF substrate adaptor module) components of the FA core complex (Swuec et al., 2017; van Twest et al., 2017). Deubiquitination was more nuanced – USP1:UAF1 could efficiently remove ubiquitin from FANCD2-^Ub^-FANCI but not FANCD2-^Ub^-FANCI-^Ub^. However if FANCD2-^Ub^-FANCI-^Ub^ is dissociated from DNA, it then becomes a USP1:UAF1 substrate (van Twest et al., 2017). In this way, USP1:UAF1 drives FANCI:FANCD2 complex toward a uniformly di-ubiquitinated state, that can only be de-ubiquitinated post-repair. We have now used this robust reconstituted system to determine if FANCI phosphorylation regulates monoubiquitination and/or deubiquitination of the complex.

## Materials and Methods

### Protein Purification

Table 1 outlines the plasmids and bacmids used in this study and their derivation. Plasmids were propagated using NEB-10-beta competent cells and purified using Monarch miniprep kits (NEB). Bacmids were generated using the Multibac system (Berger et al., 2004) and purified using alkaline lysis method followed by isopropanol precipitation and resuspension in TE.

**TABLE 1:** Plasmids and Bacmids used in this study.

**Plasmid**	**Protein**	**Selection**	**Affinity tag**	**Use**
FASTBAC1-FLAG-xFANCI	xFANCI	Ampicilin	Flag	Bacmid generation
pFASTBAC1-STREPII-xFANCD2	xFANCD2	Ampicilin	StrepII	Bacmid generation
pFASTBAC1-FLAG-hFANCD2	hFANCD2	Ampicilin	Flag	Bacmid generation
pFL-EGFP-HIS-hFANCI	hFANCI	Ampicilin	His	Bacmid generation
pFL-EGFP-FLAG-FANCB-pSPL-FAAP100-FANCL	BL100	Ampicilin, Spectinomycin	Flag	Bacmid generation
pFL-MBP-FANCC-FANCE-FANCF	CEF	Ampicilin	MBP	Bacmid generation
pGEX-KG-GST-UBE2T	UBE2T	Ampicilin	GST	*E. coli* expression

Human FANCI:FANCD2 complex and Avi-ubiquitin was purified as described in Tan et al. (2020). *Xenopus laevis* (frog) FANCI:FANCD2, human FANCB:FANCL:FAAP100, FANCC:FANCE:FANCF and UBE2T were expressed and purified as described in van Twest et al. (2017). USP1:UAF1, HA-ubiquitin and UBE1 were purchased from Boston Biochem. ATR-ATRIP was purchased from Eurofins DiscoverX. Lambda phosphatase was purchased from New England Biolabs.

#### FANCI Phosphomutants

*Xenopus laevis* StrepII-FANCD2, Flag-FANCI and human Flag-FANCI were cloned into pFastBac1 plasmid (Thermo Fisher). Expression plasmids for StrepII-FANCD2, Flag-FANCI, FANCI phosphomimic mutant (S6D) and phosphodead mutant (S6A) were previously described (Knipscheer et al., 2009; Sareen et al., 2012; van Twest et al., 2017). *Xenopus* FANCI with six codons encoding for serine (S) residues S557, S560, S566, S597, S618, and S630 (corresponding to serine residues S556, S559, S565, S595, S617, and S629 in human FANCI) mutated to encode either for aspartic acid (D) residues (FANCI6S→ D) or alanine residues (FANCI6→ A) were kindly provided by Alexandra Sobeck lab (Sareen et al., 2012). Different permutations of FANCI phosphomimic (→ D) or phosphodead (→ A) in the S3 clusters were generated as indicated in Figure 2A. Recombinant baculoviruses were generated by standard protocols (Berger et al., 2004). *Trichoplusia ni* (Hi5) insect cells were co-infected with *Xenopus* FANCI and FANCD2 viruses or infected only with human or *Xenopus* FANCI (Flag-tagged) or FANCD2 (StrepII-tagged) (MOI = 2) and harvested after 72 h. Cell pellets were washed in 1X PBS and resuspended in 9 mL Flag Lysis buffer (50 mM Tris-HCI pH 8.0, 100 mM NaCl, 1 mM EDTA, 1X mammalian protease inhibitor (Sigma-Aldrich), 10% glycerol) or Strep Lysis buffer (50 mM Tris-HCl pH 8.0, 100 mM NaCl, 1 mM EDTA, 1X mammalian protease inhibitor (Sigma-Aldrich), 10% glycerol, 20 μg/mL avidin, 1 mM DTT). Lysates were briefly sonicated and cleared by centrifugation for 45 min at 16,000 × *g*, 4°C. 1 mL M2 Flag resin (Sigma-Aldrich) or 1 mL StrepTactin Sepharose resin (VWR International) was washed with 5 CVs each of water, followed by 0.1M glycine pH 3.5 or 0.5M NaOH, and equilibrated with 10 CVs buffer A (20 mM Tris-HCl pH 8.0, 100 mM NaCl, 10% glycerol). Lysate was added to the Flag or Streptactin resin, and incubated with gentle mixing at 4°C for 2 h. Resin was washed with 10 CVs of buffer A and eluted in 1 CV buffer A with 5 μg/mL Flag peptide (Assay Matrix) or 100 μg/mL D-desthiobiotin (Sigma-Aldrich). Flag fractions containing FANCI:FANCD2 complex were pooled, and loaded onto a MiniQ anion purification column (GE) and equilibrated with buffer A. Using a gradient between buffer A and buffer B (20 mM Tris-HCl pH 8.0, 1M NaCl, 10% glycerol), FANCI:FANCD2 complex were eluted between 250 and 350 mM NaCl. The peak fractions were pooled, and assessed by SDS-PAGE. Protein concentration was determined by Nanodrop (Absorbance at 280 nm and calculated extinction coefficients).

### *In vitro* Kinase and Phosphatase Assays

Ten μg of recombinant FANCI:FANCD2, FANCI, or FANCD2 were incubated in 60 μL 20 mM Tris-HCl pH 7.4, 10 mM MgAc, 0.5 mM DTT, 0.05% Tween-20, 100 mM KCl and 0.2 mM ATP in the presence of 0.1 μg of ATR:ATRIP for 30 min at 30°C. For phosphatase experiments, 600 units of lambda phosphatase was added to reactions together with 1 mM MnCl_2_ and incubated for 30 min at 30°C prior to establishment of ubiquitination reaction.

### *In vitro* Ubiquitination and Deubiquitination Assays

Standard ubiquitination reactions contained 10 μM recombinant human AviTag-biotin-ubiquitin, 50 nM human recombinant UBE1, 100 nM UBE2T, 100 nM pUC19 plasmid or dsDNA oligonucleotide substrate, 2 mM ATP, 100 nM FANCI:FANCD2 complex wild type (WT) or ubiquitination-deficient (KR), in reaction buffer (50 mM Tris-HCl pH 7.4, 2.5 mM MgCl_2_, 150 mM NaCl, 0.01% Triton X-100). The dsDNA substrates were generated using oligonucleotide 1 (5′-ACGC TGCCGAATTCTACCAGTGCCTTGCTAGGACATCTTTGCC CACCTGCAGGTTCACCC-3′) and oligonucleotide 2 (5′-GG GTGAACCTGCAGGTGGGCAAAGATGTCCTAGCAAGGCA CTGGTAGAATTCGGCAGCGT-3′). 20 μL reactions were set up on ice and incubated at 25°C for 90 min. To perform deubiquitination assays, FANCI:FANCD2 monoubiquitination was arrested using apyrase (NEB) and 100 nM recombinant USP1:UAF1 (Boston Biochem) were added for 30 min at room temperature. Reactions were stopped by adding 10 μL NuPage LDS sample buffer and heated at 80°C for 5 min. Reactions were loaded onto 4–12% SDS PAGE and run using NuPAGE^®^ MOPS buffer and assessed by western blot analysis using Flag (Jomar Life Research) or StrepII (Abcam) antibody.

### *In gel* Proteolytic Digestion and Mass Spectrometry Analysis of FANCI Proteins

Proteins were separated by SDS-PAGE, and the band excised for manual digestion to maximize sensitivity and efficiency. Protein bands were destained and dehydrated with 500 μL acetonitrile (ACN). Subsequently, proteins were reduced with 500 μL 10 mM dithiothreitol (DTT) in 25 mM ammonium bicarbonate (NH_4_HCO_3_) at 55°C for 1 h and alkylated with 50 μL 55 mM iodoacetamide in 25 mM NH_4_HCO_3_ at room temperature for 45 min in the dark. Samples were incubated overnight with 20 μL trypsin (125 ng, 37°C). The resulted proteolytic peptides were subjected to sonication with 50 μL of 50% ACN, 5% formic acid and analyzed by mass spectrometry after concentration under vacuum to a 10–15 μL final volume. Mass spectra of digested protein gel bands were obtained on ESI-quadropole-time-of-flight mass spectrometer coupled to reverse-phase HPLC-MS/MS. The analysis program MASCOT was used to identify phosphorylation sites on FANCI.

### Biolayer Inferometry (BLItz) Kinetic Analysis

Kinetic titration series were performed in buffer F (20 mM Tris-HCl pH 8.0, 150 mM NaCl). 100 μg/ml FANCD2 or FANCI was diluted in buffer F and further diluted three times with a dilution factor of two. To measure the interaction between FANCD2 and FANCI, the association and dissociation times were 180 and 300 s, respectively, for every analyte concentration. In total, four Streptavidin sensors (ForteBio) were used to measure four different analyte concentrations in parallel, while one sensor was used to measure the buffer reference. All steps were performed at 25°C with an agitation speed of 1000 rpm. Sensorgrams were measured on a ForteBio BLItz instrument and referenced against the buffer reference signal using the Data Analysis software 7.1.0.36 (ForteBio). The sensorgrams obtained with the concentrations: 334.7, 167.3, 83.7, and 41.8 nM were fitted with the BiaEvaluation software 4.1 from Biacore using a 1:1 binding model.

### Electromobility Shift Assay (EMSA)

Sixty bp fluorescently labeled dsDNA substrates were prepared as described in van Twest et al. (2017) by annealing oligonucleotide XOm1 5′-labeled with IRDye-700 (IDTDNA) and oligonucleotide XOm1.com. 25 nM dsDNA were incubated with the indicated amounts of protein for 30 min at room temperature in a 15 μL reaction containing 6 mM Tris pH 7.5, 0.1 mM EDTA, 1 mM DTT, 6% glycerol. The reaction was resolved by electrophoresis through a 6% non-denaturing polyacrylamide gel in 1X TBE (100 mM Tris, 90 mM boric acid, 1 mM EDTA) buffer and visualized by Licor Odyssey system.

### Pull-Down Assays

Two μg of purified Flag-FANCI proteins were incubated with 2 μg of His-USP1:UAF1 (Boston Biochem) for 30 min at 25°C in 40 μL of pull-down buffer (20 mM Tris-HCl pH 8.0, 100 mM NaCl and 5% glycerol). 10 μL of TALON metal affinity resin (Takara Bio) were added to the reaction mixtures and were gently mixed for 30 min at 25°C. The resin were then washed three times with 1 mL pull-down buffer. The proteins bound to the resin were separated by SDS-PAGE and stained with Coomassie blue. The band intensities of FANCI were quantitated and visualized by Licor Odyssey system.

## Results

### Dephosphorylation of Recombinant FANCI:FANCD2 Complex Inhibits Its *in vitro* Monoubiquitination

**FIGURE 1:**
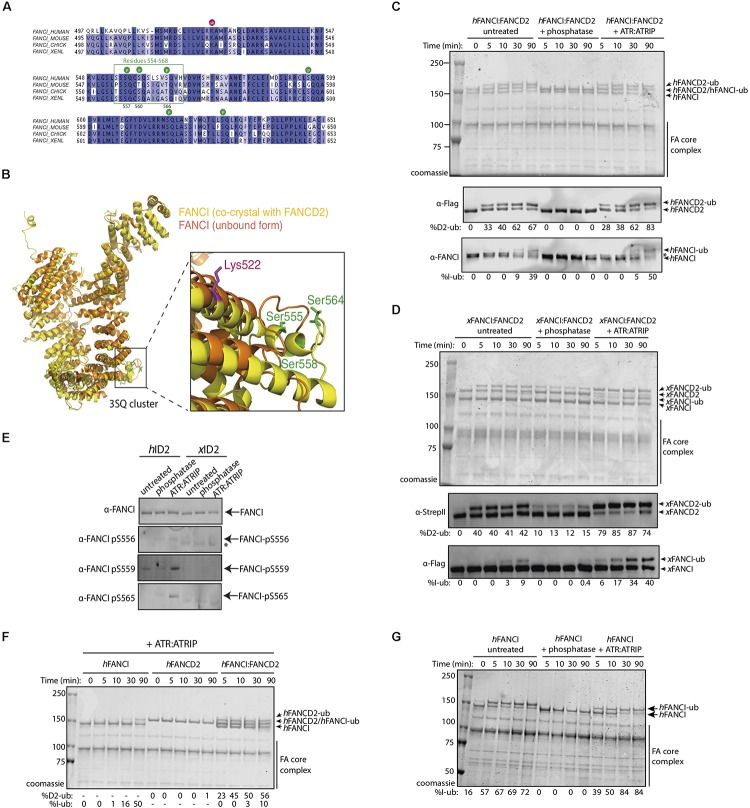
FANCI phosphorylation at serines 557, 560, and 566 are required for FANCI:FANCD2 monoubiquitination. **(A)** Sequence alignment of the FANCI SQ/TQ loop across different species including human, mouse, chicken and *Xenopus laevis* (frog). Phosphorylation sites are indicated and residues 554–569 accounting for FANCI SQ/TQ loop are boxed in green. **(B)** Superposition of FANCI unbound (orange, PDB ID 3Z51) and FANCD2-bound (yellow, PDB ID 3S4W) structures revealed little conformational differences except in the clustered SQ/TQ sites near FANCI monoubiquitination site, Lysine 522. **(C)** Coomassie stained SDS-PAGE gel showing *in vitro* ubiquitination assays of untreated, Lambda phosphatase or ATR-ATRIP kinase treated human FANCI:FANCD2 (hID2) and **(D)** Coomassie stained SDS-PAGE gel showing *in vitro* ubiquitination assays of untreated, Lambda phosphatase or ATR-ATRIP kinase treated *Xenopus* FANCI:FANCD2 (xID2) complex. **(E)** Western blot showing that human FANCI phosphosites 556, 559, and 565 are phosphorylated in ATR-ATRIP treated ID2. **(F)** Coomassie stained SDS-PAGE gel showing the ubiquitination time course of ATR:ATRIP treated isolated human FANCI, FANCD2, or FANCI:FANCD2 complex. **(G)** Coomassie stained SDS-PAGE gel showing the ubiquitination time course of untreated, phosphatase or ATR:ATRIP treated human FANCI. All data are representative of three experiments.

We determined that a percentage of recombinant FANCI purified from baculovirus infected insect cells is phosphorylated within a previously described S/TQ cluster domain (Ishiai et al., 2008 and Supplementary Figure 1). Tandem mass spectrometry (MS/MS) analysis of the purified protein revealed FANCI phosphorylation of multiple serine residues (Supplementary Figures 1B–D), including residues previously reported S557, S560, and S566 (Cheung et al., 2017). These correspond to exposed surface residues that are conserved during evolution (Figure 1A), but differ in their structure between the unbound and FANCD2-bound FANCI (Figure 1B). ATR is the kinase predicted to phosphorylate S557, S560, and S566 in FANCI.

To determine the contribution of the identified phosphorylation sites to FANCD2:FANCI monoubiquitination, we performed an extended phosphatase treatment and then performed *in vitro* ubiquitination reactions using recombinant FA core complex. In both human (Figure 1C) and *Xenopus* FANCI:FANCD2 (Figure 1D), treatment with λ-phosphatase almost completely eliminated the monoubiquitination of FANCI, and significantly reduced the monoubiquitination rate of FANCD2. We show that addition of recombinant ATR-ATRIP kinase can restore the mono-ubiquitination levels in these preparations. As such, we observed the reappearance of positive bands when using phosphospecific antibodies raised against these three residues, only in ATR-ATRIP treated human FANCI:FANCD2 samples (Figure 1E). Together using phospho-specific antibodies and mass spectrometry analysis (Supplementary Figure 1), we show that three FANCI serine residues are the substrates of ATR kinase, and are required for optimal monoubiquitination of FANCI and FANCD2.

Free hFANCI, and to some extent hFANCD2, can also be substrates for monoubiquitination by the FA core complex (Longerich et al., 2009; Hodson et al., 2014). We therefore tested the requirement for ATR-phosphorylation in mediating the monoubiquitination of each subunit in isolation. Surprisingly, we found that human FANCI is monoubiquitinated faster in a free state, than when it is in complex with FANCD2, while free FANCD2 remains unubiquitinated (Figure 1F). Furthermore, free FANCI is ubiquitinated at the same fast rate as ATR:ATRIP treated FANCI, but is very slow when dephosphorylated (Figure 1G). This result suggests phosphorylation of FANCI specifically mediates its effects on FANCI even when it is not bound to FANCD2.

### FANCI S6D Phosphomimic Mutant Binds to FANCD2 With Reduced Affinity

**FIGURE 2:**
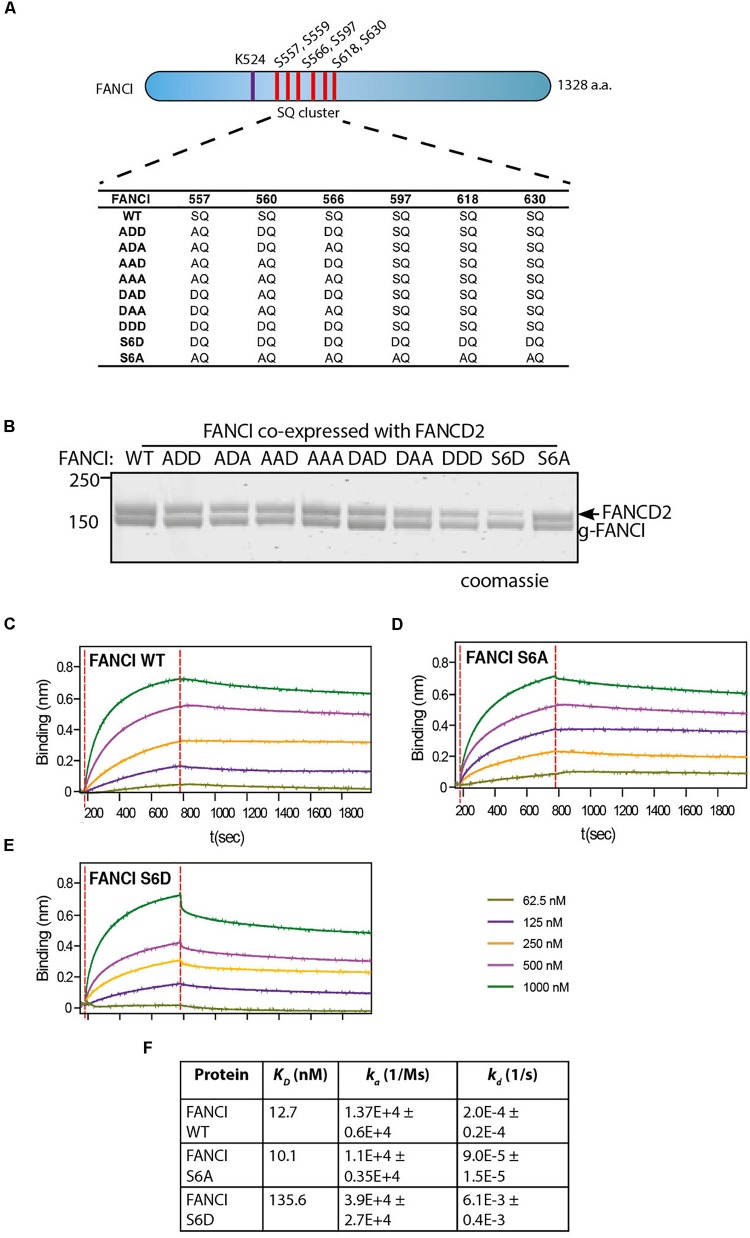
FANCI phosphomimic mutant at six serine sites but not three serine sites dissociates ID2 complex. **(A)** Schematic of *Xenopus* FANCI, indicating the six phosphorylation sites within the SQ cluster region. The phosphorylation sites serine 557, 560, 566, 597, 618, and 630 were replaced either by aspartate (DQ) or alanine (AQ). The FANCI mono-ubiquitination site lysine 524 is also shown. **(B)** Coomassie stained SDS-PAGE of Flag-ID2 complex with StrepII-FANCD2 co-expressed with various flag-FANCI phosphomutants. **(C–E)** Biolayer inferometry (BLItz) sensorgrams obtained using StrepII-FANCD2-loaded biosensors in 20 ng/mL solution, with red dotted lines indicating the start of binding (left) and dissociation (right) phases. Biosensors loaded with StrepII-FANCD2 were incubated with different concentrations of FANCI wild type (WT), S6A or S6d mutants, as indicated to generate a series of sensorgrams. **(F)** Summary of dissociation constant (K_*D*_), association (K_*a*_), dissociation (K_*d*_) rate constants obtained from the sensorgrams. Data are representative of four experiments.

Three S/TQ residues, in additional to those described above, were also predicted to be conserved ATR sites in human (Ishiai et al., 2008) and *Xenopus* FANCI (Sareen et al., 2012). However, these predictions were made prior to the derivation of the FANCI:FANCD2 crystal structure. The residues, S597, S618, and S630 turned out to be completely buried in the solenoid structure of FANCI and unlikely to be accessible to any kinase without complete unfolding of the protein structure. None-the-less, the xFANCI-S6D (6 serines changed to aspartate “phosphomimic”) and xFANCI-S6A (6 serines changed to alanine “phosphodead”) mutants were subsequently used to hypothesize that FANCI phosphorylation leads to the dissociation of FANCD2 and FANCI (Sareen et al., 2012). In our *in vitro* studies, ATR phosphorylation of FANCI did not cause dissociation of the FANCD2:FANCI heterodimer. This led us to suspect that phosphomutant S6A and phosphomimic S6D amino acid substitutions at these residues could be affecting the integrity of the FANCI structure and/or heterodimer formation. We examined xFANCI-S6D (where all 6 serines in the SQ cluster are changed to phosphomimic aspartate residues, Figure 2A) and confirmed that it has reduced co-purification with xFANCD2 (Figure 2B and Supplementary Figure 2). This was a result of an order of magnitude decrease in the affinity of xFANCI-S6D compared to WT, as measured by biolayer inferometry (Figures 2C,D,F). xFANCI-S6A, showed normal co-purification with xFANCD2 and a similar affinity to FANCD2 as WT (Figures 2B,E).

We never observed S597, S618, and S630 to be phosphorylated in our mass spectrometry studies of recombinant ATR-ATRIP phosphorylated xFANCI or hFANCI protein, and a recent publication failed to identify phosphorylation of these sites in endogenous human FANCI (Cheung et al., 2017). We therefore conclude that the S557/S560/S566 (the 3SQ cluster) is likely to be the functional ATR-phosphorylation cassette in FANCI. So, we repeated the co-purification experiments using phospho-mimic/dead mutants of the 3SQ cluster in various permutations to determine if affinity changes were also observed when these sites only are altered. Unlike for xFANCI-S6D, each of the xFANCI 3SQ mutants bound to xFANCD2 in a 1:1 ratio (Figure 2B and Supplementary Figure 2). We conclude that ATR-regulation of FANCI on the surface 3SQ cluster does not lead to dissociation of the heterodimer, and likely plays some other role in regulation of FANCD2 and/or FANCI monoubiquitination.

### Phosphorylation of FANCI in the S3Q Cluster Promotes FANCI:D2 Monoubiquitination by Increasing Its Retention on DNA

**FIGURE 3:**
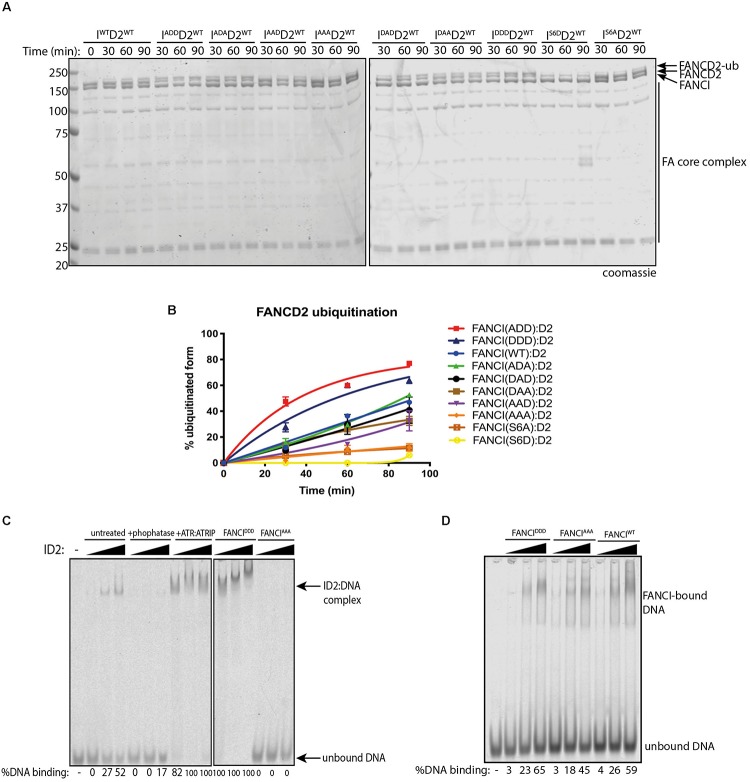
FANCI phosphorylation in the S3Q cluster regulates FANCD2 ubiquitination by increasing its retention on DNA. **(A)** *In vitro* ubiquitination assays of various FANCI-phosphomutant:FANCD2 complexes showing that phosphomimic mutants present higher ubiquitination rate than phosphodead mutant. **(B)** Quantification showing the percentage of FANCD2 monoubiquitination using various FANCI phosphomutants. Data are representative of three experiments. **(C)** Electro mobility shift assay (EMSA) gels showing 100. 200 and 400 nM of untreated, phosphatase or ATR: ATRIP treated ID2 (WT. DDD or AAA) complexes and **(D)** isolated FANCI (WT. DDD or AAA) proteins in the presence of 25 nM IRDye-700 dsDNA. The percentage of protein binding to DNA was calculated and shown under native PAGE gels. Data are representative of three experiments.

To test if phospho-mimic or phospho-dead mutants within the xFANCI 3SQ cluster affect FANCI:FANCD2 ubiquitination, we performed *in vitro* ubiquitination assays of FANCI:FANCD2 WT and each of the FANCI-3SQ-mutants using recombinant FA core complex proteins. Four of the xFANCI phosphomimic mutants (ADD, ADA, DAD, and DDD) showed faster monoubiquitination rates than for WT xFANCI:FANCD2 complex. In particular, the FANCI-ADD and -DDD variants stimulated the highest rate of xFANCD2 monoubiquitination. Conversely, two of the FANCI-3SQ mutants (AAD and AAA) and both of the FANCI-6SQ mutants (S6D and S6A) showed lower xFANCD2 monoubiquitination than WT (Figure 3A). These results demonstrate that phosphomimic mutations in S560 together with S565 are sufficient to maximally stimulate monoubiquitination of FANCI:FANCD2 complex by the FA core complex (Figure 3B).

Previous studies have shown that optimal FANCI:FANCD2 monoubiquitination requires the association of the complex with DNA (Sato et al., 2012; Longerich et al., 2014; van Twest et al., 2017) leading us to speculate that phosphorylation may increase the affinity of FANCI:FANC2 complex to DNA. To examine this hypothesis, we performed electromobility shift assays (EMSAs). Remarkably, the DNA binding measured by EMSA shift was greatly reduced in lambda-phosphatase treated FANCI:FANCD2 complex or FANCI AAA mutant compared to control (Figure 3C). DNA binding was not only restored after phosphorylation by ATR and in the FANCI DDD mutant, but occurred at significantly lower FANCI:FANCD2 concentration (i.e., higher affinity). We further tested the association of isolated FANCI with DNA and found that phosphatase treatment also caused a reduction in DNA binding that was restored by ATR-kinase treatment (Figure 3D). These results are consistent with ATR-phosphorylation creating a higher affinity of FANCI (and associated FANCD2) for DNA binding, leading to a greater stimulation of monoubiquitination of FANCD2 by the FA core complex.

### FANCI Phosphorylation or Phosphomimics Also Protect FANCD2 From Deubiquitination

**FIGURE 4:**
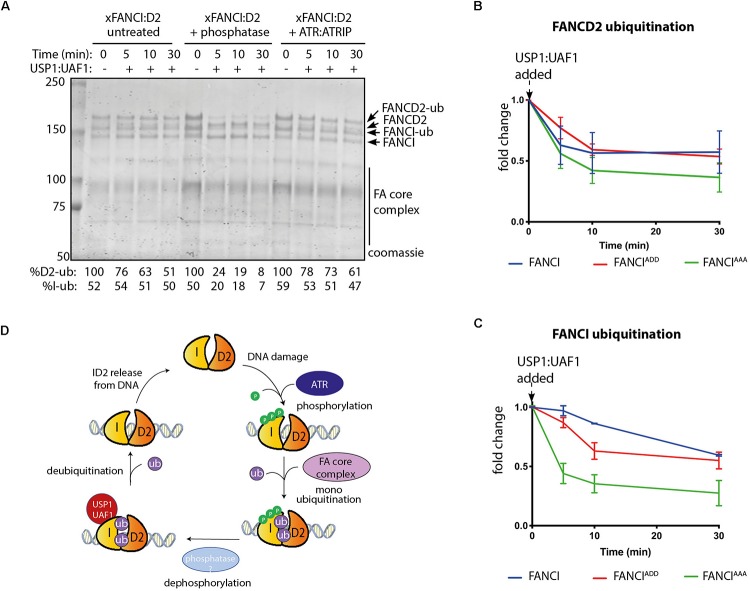
FANCI dephosphorylation as a switch to turn off FANCLFANCD2 deubiquitination. **(A)** Coomassie stained SDS-PAGE showing the deubiquitination time course of untreated, phosphatase and ATR-ATRIP treated *Xenopus* FANCLFANCD2 complexes. **(B)** Graphical representation of FANCD2 or **(C)** FANCI ubiquitination fold change after USP1:UAF1 is added within the FANCI^*WT*^:FANCD2, FANCI^*ADD*^:FANCD2 and FANCI^*AAA*^:FANCD2 complexes (representative experiment of *n* = 3). **(D)** Model of the role of FANCI phosphorylation in regulating FANCL:FANCD2 monoubiquitination and deubiquitination.

Published evidence from cell-based studies suggests phosphorylation of FANCI by ATR could also regulate FANCD2 deubiquitination (Cheung et al., 2017). To examine whether FANCI deubiquitination activity by USP1:UAF1 is regulated by the 3SQ cluster, we performed *in vitro* deubiquitination reactions where xFANCI^Ub^:xFANCD2^Ub^ was treated with lambda phosphatase after ubiquitination. Most of the xFANCI^Ub^ was resistant to deubiquitination in the native state, but dephosphorylation accelerated the deubiquitination. xFANCD2^Ub^ was also significantly faster. Re-phosphorylation of the xFANCI^Ub^:xFANCD2^Ub^ restored the slower rate of USP1:UAF1-mediated deubiquitination (Figure 4A). This was due to a direct effect of ATR phosphorylation on FANCI because xFANCD2 and FANCI-(AAA)^Ub^ was also deubiquitinated significantly faster than xFANCI^Ub^ or xFANCI-(ADD)^Ub^. Overall, the rate of FANCD2 deubiquitination when bound to FANCI-ADD or WT-FANCI is fourfold slower than for FANCI-AAA (Figures 4B,C).

Taken together, our results suggest that ATR-mediated FANCI phosphorylation both promotes monoubiquitination and inhibits deubiquitination of FANCD2.

## Discussion

In humans, ATR kinase strongly influences the activation of the Fanconi Anemia DNA repair pathway (Andreassen et al., 2004). In particular, FANCI phosphorylation by ATR kinase was proposed to be an “on switch” for FANCD2 monoubiquitination (Ishiai et al., 2008; Cheung et al., 2017). The present work uncovers additional evidence that it is the direct phosphorylation of FANCI by ATR that influences the biochemical rate of FANCD2 ubiquitination *and* deubiquitination to reveal fundamental insights into how FANCD2-monoubiquitination is maintained at sites of DNA damage.

We found that three sites on the surface of FANCI, serines 557, 560 and 565, are the main targets of recombinant ATR kinase *in vitro*. Phosphomimic mutations at these sites, create a charge effect similar to true phosphorylation, and stimulated the *in vitro* monoubiquitination of FANCD2. However, we did not see this effect when we used a mutant called S6D. We propose that the additional three serines (S597, S618, and S630) mutated in this variant may cause structural malformation of FANCI, given their location within α-helices of the solenoid structure of the protein. As such, FANCI-S6D only binds weakly to FANCD2 and does not stimulate its mono-ubiquitination. This finding supports the *in vivo* cell-based studies of Cheung et al. (2017) where only these three phospho-serines were identified in mass-spectrometry based analysis, but does not support the concept of FANCI dissociation from FANCD2 after phosphorylation or monoubiquitination (Sareen et al., 2012). The actions of ATR kinase on the FANCI:FANCD2 complex are therefore likely to be restricted to these sites associated with DNA damage response.

We previously showed that DNA-PKcs, a kinase related to ATR, could phosphorylate FANCI but could not stimulate FANCD2 monoubiquitination (van Twest et al., 2017). This indicates that DNA-PKcs is a poor substitute for ATR, or that it cannot catalyze the correct combination of FANCI phosphorylation events necessary for activation. However, it’s possible that other kinases could further modulate the temporal and/or spatial localization of FANCD2 and FANCI monoubiquitination *in vitro* or in cells. For example, Casein Kinase 2 phosphorylation of FANCD2 at a cluster of serines between residues 882–898 inhibits its DNA association and subsequent monoubiquitination (Lopez-Martinez et al., 2019), while ATM phosphorylates many residues in FANCD2 independent of monoubiquitination, but only during S-phase and after ionizing radiation (Taniguchi et al., 2002b; Ho et al., 2006). Other kinases with an important role in DNA replication and DNA damage response have yet to be explored, although several such as Chk1 and CDK also phosphorylate subunits in the FA core complex (Deans et al., 2006; Wang et al., 2007). Kinases may also regulate enigmatic dimerization and monoubiquitination-independent functions of FANCI and FANCD2, that appear to be necessary only after certain types DNA damage (Sareen et al., 2012; Chaudhury et al., 2013; Chen et al., 2016). A thorough biochemical investigation of the complex interplay of known and unknown kinases in the FA pathway is likely to be fruitful.

Deubiquitination of FANCD2 and FANCI appears to be as important as ubiquitination, in the regulation of the Fanconi Anemia pathway. There are at least two reasons for this: (1) it prevents the retention of FANCD2 at spurious, non-repair sites in the nucleus and (2) it allows completion of DNA repair (Cohn et al., 2009; Tan and Deans, 2017). A major function of FANCI^Ub^ appears to be the prevention of FANCD2^Ub^ deubiquitination, but only while it is DNA associated. Our data show that a phosphomimic mutant of FANCI is further protective to FANCD2^Ub^ deubiquitination. Supporting our results, Cheung et al. (2017) showed that FANCI phosphomimic mutant at serine 560 and 566 prevents deubiquitination of FANCD2 *in vivo* (Cheung et al., 2017). Together, these results suggest that FANCI dephosphorylation might be required for efficient FANCI:FANCD2 deubiquitination. Additional studies will be required to understand the dephosphorylation of FANCI and whether it alters FANCD2 ubiquitination in cells. CTDP1 and PTEN are phosphatases known to act in the FA pathway, although neither have been demonstrated to act directly on FANCI (Vuono et al., 2016; Hu et al., 2019). Our *in vitro* reconstitution system will be useful for answering mechanistic questions regarding FANCI dephosphorylation and for identifying potential phosphatases that regulate the FA pathway.

FANCD2 monoubiquitination by FA core complex and deubiquitination by USP1:UAF occurs at steady state levels associated with ongoing replication (Taniguchi et al., 2002a; Liang et al., 2016). We propose that upon replication fork stalling, ATR kinase is activated, with locally high concentrations of active ATR promoted by the activity of FANCM:FAAP24:hCLK2 complex (Collis et al., 2008). ATR phosphorylates the FANCI-3SQ cluster. From structural models, phosphorylation of these residues are predicted to increase the surface area of interaction between FANCD2 and FANCI by at least twenty-five percent (Joo et al., 2011), which likely explains the increased affinity of the complex on DNA. This would stabilize the complex at damage sites, leading to its increased monoubiquitination by the FA core complex, which is also localized by FANCM (Deans and West, 2009). We also predict that a conformational change induced by phosphorylation of the S3Q cluster alters the association of the adjacent SLIM domain of FANCI with USP1:UAF1, as a mechanism for the observed reduction in USP1:UAF1 binding in S3Q phosphomimic proteins. Therefore, deubiquitination is prevented until repair is completed and the ATR kinase is deactivated (Figure 4D).

Given the critical importance of FANCI as an ATR target, the phosphorylation state of the FANCI-3SQ cluster may be an appropriate biomarker of the effectiveness of several classes of ATR kinase inhibitor currently in clinical trial for the treatment of cancer (Lecona and Fernandez-Capetillo, 2018). Our work also suggests that direct modulation of FANCI phosphorylation plays a twofold role in stabilizing FANCD2 monoubiquitination, with relevance to understanding and treating both Fanconi anemia and cancer.

## Data Availability Statement

The datasets generated for this study are available on request to the corresponding author.

## Author Contributions

WT performed protein purification, biolayer inferometry, ubiquitination, deubiquitination, and DNA binding assays. ST performed deubiquitination assays. VM generated FANCI phosphomutants and assisted with protein purifications. WT and AD wrote the manuscript and designed experiments for this article.

## Conflict of Interest

The authors declare that the research was conducted in the absence of any commercial or financial relationships that could be construed as a potential conflict of interest.
