# Supplementary material for: ATR-Mediated FANCI Phosphorylation Regulates Both Ubiquitination and Deubiquitination of FANCD2
Source: Front Cell Dev Biol. 2020 Feb 4;8:2. doi: 10.3389/fcell.2020.00002 (PMC7010609; doi:10.3389/fcell.2020.00002)
Supplement: Supplementary file 1 [file Data_Sheet_1.PDF]

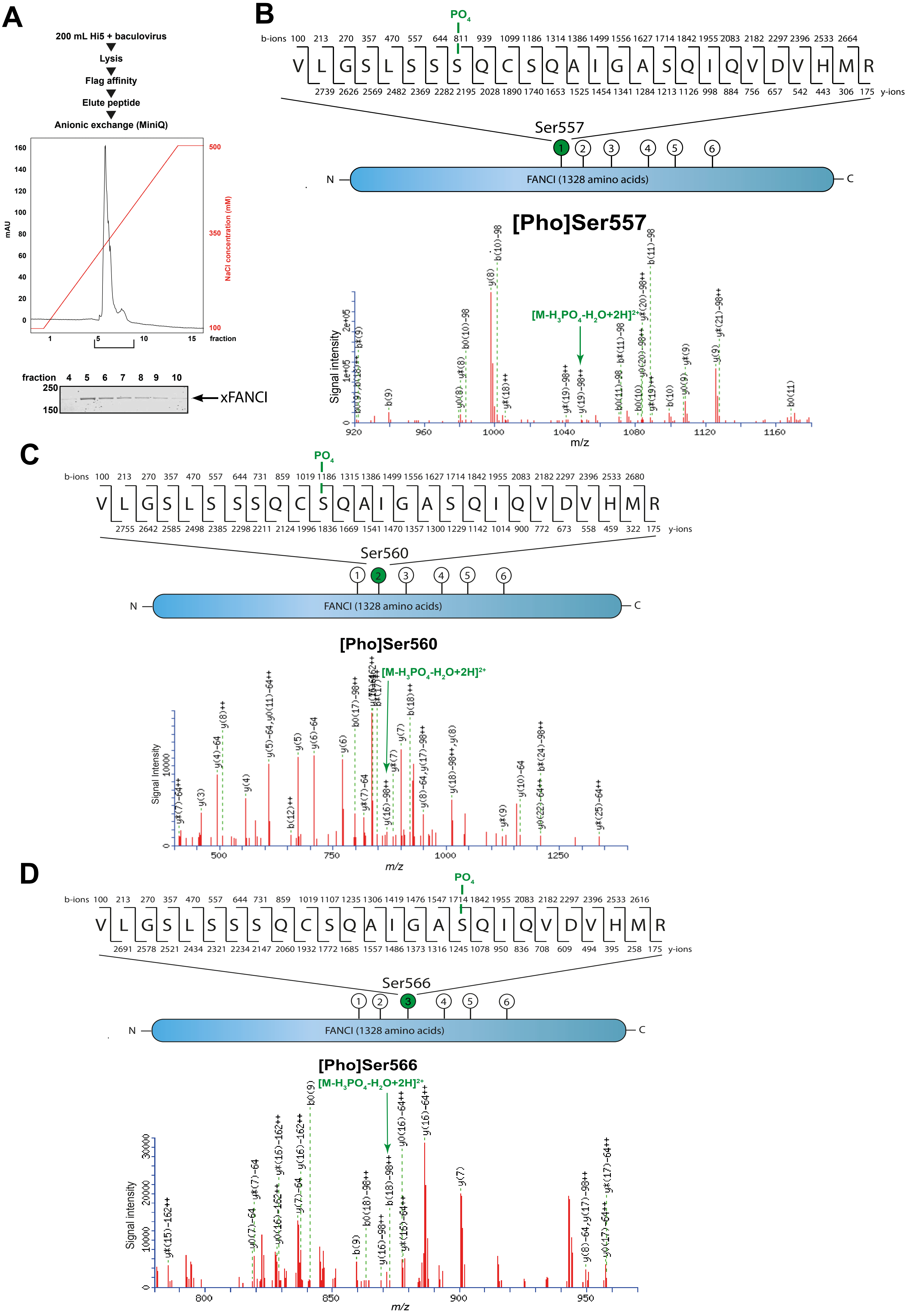
A
200 mL Hi5 + baculovirus
Lysis
Flag affinity
Elute peptide
Anionic exchange (MiniQ)
mAU
NaCl concentration (mM)
fraction
xFANCI
B
PO4
b-ions 100 213 270 357 470 557 644 811 939 1099 1186 1314 1386 1499 1556 1627 1714 1842 1955 2083 2182 2297 2396 2533 2664
V L G S L S S S Q C S Q A I G A S Q I Q V D V H M R
2739 2626 2569 2482 2369 2282 2195 2028 1890 1740 1653 1525 1454 1341 1284 1213 1126 998 884 756 657 542 443 306 175 y-ions
Ser557
N
FANCI (1328 amino acids)
C
[Pho]Ser557
[M-H3PO4-H2O+2H]2+
Signal intensity
m/z
C
PO4
b-ions 100 213 270 357 470 557 644 731 859 1019 1186 1315 1386 1499 1556 1627 1714 1842 1955 2083 2182 2297 2396 2533 2680
V L G S L S S S Q C S Q A I G A S Q I Q V D V H M R
2755 2642 2585 2498 2385 2298 2211 2124 1996 1836 1669 1541 1470 1357 1300 1229 1142 1014 900 772 673 558 459 322 175 y-ions
Ser560
N
FANCI (1328 amino acids)
C
[Pho]Ser560
[M-H3PO4-H2O+2H]2+
Signal Intensity
m/z
D
PO4
b-ions 100 213 270 357 470 557 644 731 859 1019 1107 1235 1306 1419 1476 1547 1714 1842 1955 2083 2182 2297 2396 2533 2616
V L G S L S S S Q C S Q A I G A S Q I Q V D V H M R
2691 2578 2521 2434 2321 2234 2147 2060 1932 1772 1685 1557 1486 1373 1316 1245 1078 950 836 708 609 494 395 258 175 y-ions
Ser566
N
FANCI (1328 amino acids)
C
[Pho]Ser566
[M-H3PO4-H2O+2H]2+
Signal intensity
m/z

Supplementary Figure 1. Determination of the phosphorylation state of R\recombinantly purified FANCI from Hi5 insect cells (A) Purification scheme and chromatograph of the final purification step. A bracket indicates the elution position of FANCI (fractions 5 to 10). Coomassie staining of FANCI protein in Mini Q fractions 4 to 10. (B-D) Mass spectrometric determination of phosphorylation sites on purified recombinant FANCI. The relative positions of the mapped sites on FANCI are illustrated on top. These sites correspond to Ser557, Ser560, Ser566, Ser597, Ser618 and Ser630. Shown on the bottom is the tandem mass (MS/MS) spectrum of a phosphopeptide derived by collision-induced dissociation of the (M+2H)2+ precursor, m/z 2837. Fragment ions in the spectrum represent mainly single-event preferential cleavage of the peptide bonds resulting in the sequence information recorded simultaneously from both the N- and C termini (b- and y-type ions, respectively) of the peptide. This spectrum was computer-searched with the MASCOT program (19) and was matched to a FANCI peptide with additional mass from a phosphate residue (sequence shown on the top). With six potential sites of phosphorylation, the correct assignment Ser557 (B), Ser560 (C) and Ser566 (D) were determined based on the presence of ions derived by cleavage at the Ser-Gln (SQ) peptide bond.

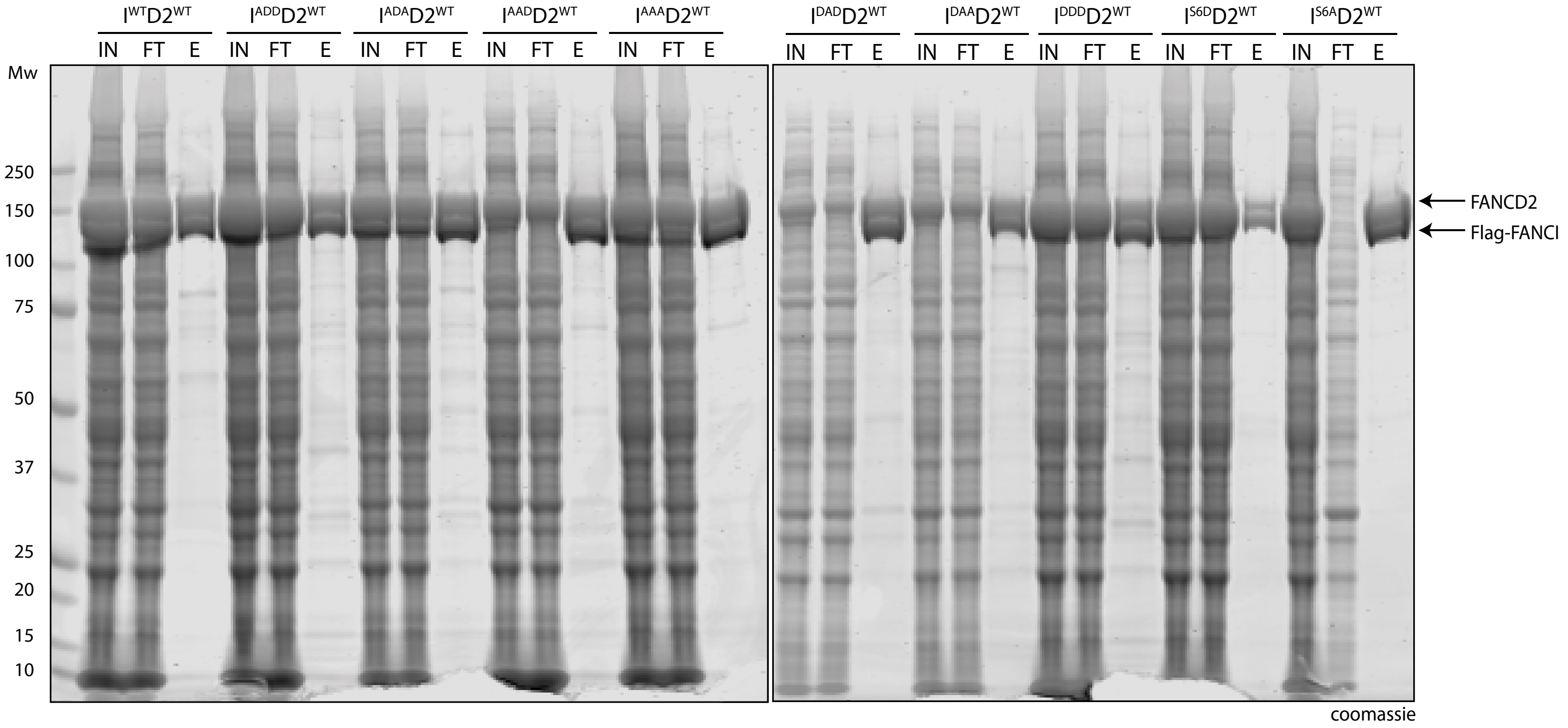

Supplementary Figure 2. Coomassie stained SDS-PAGE of Flag purification of Flag-FANCI:FANCD2 complex of various phospho-mutants

Supplementary Figure 2
